# Hypodermic Chloroform

**Published:** 1874-11

**Authors:** 


Deep Injection of Chloroform for the Relief of Douloureux. Dr. Roberts Bartholow communicates to *The Practitioner* (June, 1874,) an account of several cases of this painful affection treated successfully by hypodermic injections of chloroform.

The infra-orbital branch of the nerve was the seat of the *tic* in the cases reported, and Dr. B.'s operation consisted in passing the needle under the upper lip in the direction of and near to the infra-orbital foramen, and then injecting from ten to twenty minims of pure chloroform. Considerable pain at first ensues, followed by a feeling of numbness and anæsthesia of the parts into which the chloroform diffuses. A puffy swelling quickly forms at the site of the injection, and an induration which lasts for several days follows. One very severe case operated upon in this manner gained relief from one injection covering a period of months.—*Phil. Med. Times.*

